# The gold complex auranofin: new perspectives for cancer therapy

**DOI:** 10.1007/s12672-021-00439-0

**Published:** 2021-10-20

**Authors:** Farah H. Abdalbari, Carlos M. Telleria

**Affiliations:** 1 Experimental Pathology Unit, Department of Pathology, Faculty of Medicine and Health Sciences, McGill University, Montreal, QC Canada; 2 Cancer Research Program, Research Institute, McGill University Health Centre, Montreal, QC Canada

**Keywords:** Auranofin, Cancer, Thioredoxin reductase, Immunogenic cell death, Cisplatin

## Abstract

Advanced stages of cancer are highly associated with short overall survival in patients due to the lack of long-term treatment options following the standard form of care. New options for cancer therapy are needed to improve the survival of cancer patients without disease recurrence. Auranofin is a clinically approved agent against rheumatoid arthritis that is currently enrolled in clinical trials for potential repurposing against cancer. Auranofin mainly targets the anti-oxidative system catalyzed by thioredoxin reductase (TrxR), which protects the cell from oxidative stress and death in the cytoplasm and the mitochondria. TrxR is over-expressed in many cancers as an adaptive mechanism for cancer cell proliferation, rendering it an attractive target for cancer therapy, and auranofin as a potential therapeutic agent for cancer. Inhibiting TrxR dysregulates the intracellular redox state causing increased intracellular reactive oxygen species levels, and stimulates cellular demise. An alternate mechanism of action of auranofin is to mimic proteasomal inhibition by blocking the ubiquitin–proteasome system (UPS), which is critically important in cancer cells to prevent cell death when compared to non-cancer cells, because of its role on cell cycle regulation, protein degradation, gene expression, and DNA repair. This article provides new perspectives on the potential mechanisms used by auranofin alone, in combination with diverse other compounds, or in combination with platinating agents and/or immune checkpoint inhibitors to combat cancer cells, while assessing the feasibility for its repurposing in the clinical setting.

## Auranofin: a brief history

**Fig. 1:**
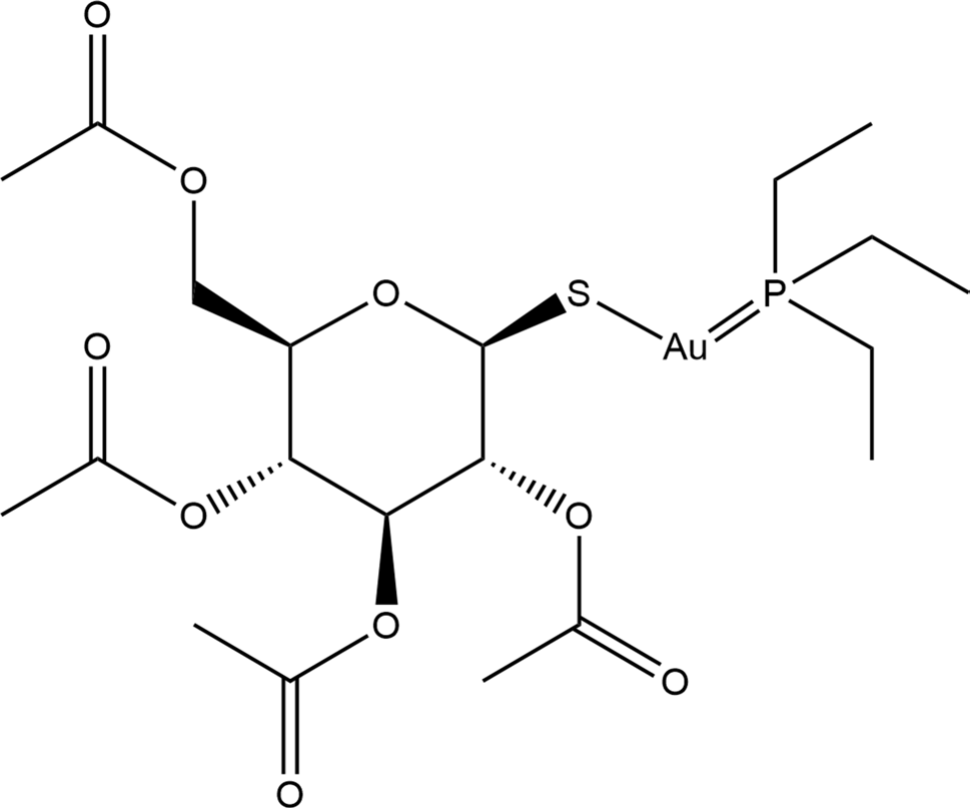
Structural formula of auranofin. The molecule consists of a monomeric linear complex with triethylphosphine and thiolate moieties bounded to an Au (I) center [10]. The molecule was adapted from the structure published in the CheBI database [11] using ChemDraw software

Auranofin is a gold (I)-containing compound that was approved by the United States Food and Drug Administration in 1985 as a primary treatment against rheumatoid arthritis [1]. Gold compounds were early on used to treat tuberculosis and other diseases including syphilis and even psychiatric conditions [2]. Originally, gold compounds were selected as a form of treatment against rheumatoid arthritis because the disease was first thought to be caused by mycobacterium tuberculosis. The original gold compounds were highly toxic, until (2,3,4,6-tetra-*O*-acetyl-1-thio-β-d-glucopyranosato-*S*) (triethylphosphine) gold (I) was synthesized: it was termed auranofin (Fig. 1) [1, 3]. This drug is a metal gold (I) complex that acts as a prodrug, which is metabolized to their pharmacologically active derivatives after being given to the patient. The drug complex consists of two parts, a water-soluble aurothioglucose entity with a sulfur donor group, and a phosphine ligand that provides lipophilicity. The drug undergoes irreversible oxidation of the thioglucose tetraacetate accompanied by hydrolysis, leading to progressive deacetylation forming two main active forms: a triethylphosphinenegold (I) cation, and a gold (I) thioglucose species, with a variable number of acetyl groups [4]. Due to its chemical nature, auranofin has high selectivity for sulfur and selenium ligand proteins with exposed free cysteines, which are modified through coordination of the gold-triethylphosphine fragment [5, 6]. For instance, classic proteomic strategies and mass spectrometry-based redox proteomics demonstrated that auranofin affects proteins primarily involved in cell redox homeostasis, and oxidizes over five hundred cysteine-containing peptides [7, 8]. The drug is administered orally for the treatment of active progressive rheumatoid arthritis, and is commercialized under the brand name of Ridaura [9].

Rheumatoid arthritis is a systemic, chronic, polyarticular inflammatory autoimmune disease that primarily targets the joints and causes increased swelling and inflammation in hands and feet. The disease has no cure and treatment goals are to reduce the pain and slow down further joint damage. Acute symptoms of the disease can be treated with nonsteroidal anti-inflammatory drugs (NSAIDs), such as acetylsalicylate, naproxen or ibuprofen, to alleviate pain, swelling and decrease inflammation. Corticosteroids are also used as more potent anti-inflammatory medications for acute symptoms yet with greater side effects. For the second line or chronic treatment, the gold complex auranofin was originally used to ameliorate the progression of the disease; however, the drug was displaced by a plethora of compounds jointly termed disease-modifying antirheumatic drugs or DMARDs including methotrexate, hydroxychloroquine, and sulfasalazine, all considered as synthetic immunosuppressive DMARDs. In addition, newer medications termed biological DMARDs are currently used, in combination with methotrexate, and consist mainly of inhibitors of cytokine targets such as TNF inhibitors, IL-6 and IL-1 receptor inhibitors, and JAK signaling inhibitors that prevent the recruitment of cells that cause a pro-inflammatory cascade associated with rheumatoid arthritis [12–16].

The major mechanism of action of auranofin against rheumatoid arthritis is to manage the autoimmune response via the inhibition of immune cell infiltration to the site of inflammation. This occurs via the suppression of T cell mitogenesis and macrophage cytotoxicity. Of interest, auranofin was shown to inhibit leukocytes and macrophages in a greater extent on cells drawn from patients with active rheumatoid arthritis [17]. Auranofin appears to be effective in decreasing lysosomal enzyme release; for instance, the gold in auranofin is concentrated within lysosomes of tissue macrophages. These lysosomal bodies were found in chondrocytes, synovial membrane cells, and the subsynovial macrophages of the joint. The lysosomal bodies containing soluble gold compounds have a particular morphological pattern, and have been early on termed ‘aurosomes’ [18]. Auranofin has been shown to be a potent inhibitor of lysosomal enzymes [19], including β-glucuronidase, lysozyme, acid phosphatase, and cathepsin, which have been shown to be enriched in the synovial fluid of patients with rheumatoid arthritis [20]. Auranofin also indirectly inhibits the secretion of pro-inflammatory cytokines, such as IL-8 and IL-6 from macrophages and monocytes, by inhibiting the NF-kB signaling pathway [21–24]. Furthermore, auranofin suppresses the immune response via the inhibition of antibody-dependent complement lysis and chemotaxis, or migration of monocytes in the bloodstream to phagocytose the arthritic cells [19]. Auranofin was also reported to inhibit prostaglandin synthesis, suppress the stimulatory action of prostaglandins [20], inhibit platelet aggregation [25], and inactivate the complement [26]. These functions and more recent investigations [27] demonstrate the role of auranofin against chronic inflammation, which poses it as a strong agent against various diseases, such as cancer, in which pro-inflammatory reactions readily occur and promote all stages of tumorigenesis within the tumor microenvironment [28].

## Auranofin is a pro-oxidative agent targeting the thioredoxin reductase system

A primary mechanism of action discovered for auranofin is acting as a pro-oxidant agent by disrupting the reduction/oxidation (redox) system within the cell. This occurs via the inhibition of thioredoxin reductases (TrxRs) represented by two selenoenzyme isoforms, a cytoplasmic form or TrxR1, and a mitochondrial form or TrxR2. They act as antioxidants regulating the levels of reactive oxygen species (ROS), thus protecting the cells from the deadly consequences of damage due to oxidative stress [16, 29].

TrxRs have a redox active selenocysteine moiety [2, 30]; mass spectrometry studies suggest that TrxRs bind to approximately four triethylphosphinenegold (I) cation (AuPet3+) fragments, while biochemical assays show that the gold compound greatly alters the active selenocysterine site of the enzymes [31–35]. It seems that the cytotoxicity of auranofin is related to the inhibition of both, cytoplasmic TrxR1 and mitochondrial TrxR2 [31]. TrxRs act in a nicotinamide adenine dinucleotide phosphate (NADPH)-dependent manner, by transferring electrons from NADPH to the active disulfide site on the oxidized Trx protein [36, 37]. The interaction between the active site dithiol in reduced Trx and oxidized cysteines of many proteins induces the process of thiol/disulfide exchange reaction to form an oxidized Trx; in this manner, Trx catalyzes the reduction of ROS from oxidized cysteines of proteins and in the process, Trx itself becomes oxidized [38–40].

The presence of cytoplasmic Trx1 in its reduced state is critical due to its multifunctional role in cellular processes such as inhibition of cell death via the binding of reduced Trx1 to apoptosis signalling kinase 1 (ASK1). The oxidation of Trx1 by ROS leads to the dissociation of Trx1 from the pro-apoptotic molecule ASK1 thus activating ASK1-induced cell death via the c-JUN N-terminal kinase (JNK) and p38 MAP kinase pathways in the cytoplasm [29]. Trx1 is also involved in cell growth and proliferation by inhibiting phosphatase and tensin homolog (PTEN) and increasing AKT activity [41]. Finally, it was shown that Trx1 translocates from the cytoplasm to the nucleus and activates a number of transcription factors including NF-kB and tumor suppressor p53 [39]. Based on these cellular effects of Trx1, it is expected that the function of auranofin in inhibiting TrxR1 would lead to increased ROS, promotion of ASK-induced apoptosis, and blockage of cell growth, proliferation, and survival due to reduced AKT activity and NF-kB- and p53-mediated transcription.

## Thioredoxin reductase 1 is over-expressed in cancer cells in association with decreased patient survival

The overexpression of TrxR1/Trx1 has been shown in breast, ovarian, colorectal, lung, pancreatic, and gastric cancers [40, 42–45]. Of interest, TrxR1 overexpression was detected in aggressive mammary tumors, in comparison with non-aggressive tumors [46]. Another interesting analysis was completed using an online public genomic database that indicated an association between the expression of the TrxR1 isoform and ovarian cancer prognosis. A Kaplan Meir survival curve was generated on a sample of over one-thousand ovarian cancer patients, which were grouped separately based on high and low TrxR1 expression; results showed that there was a significant difference in the overall survival of patients in the high TrxR1 expression group in comparison to the low TrxR1 expression group; a hazard ratio of 1.5 indicated that patients with a high TrxR1 expression have 1.5 times chances of reaching a fifty percent overall survival in a shorter time than patients in the low TrxR1 expression group [40]. This signifies that TrxR1 overexpression is closely associated with tumorigenesis and poor prognosis of the disease. Consequently, this upregulation makes auranofin an interesting anti-cancer molecule as target of the TrxR1/Trx1 system [40]. The up-regulation of TrxR1/Trx1 allows the maintenance of a normal redox balance in the context of high metabolism of the cancer cell, which because of its nature produces higher ROS levels and maintains a thither antioxidant control than non-cancer cells. The upregulation of TrxR1 occurs through the activation of Nrf2, a redox-sensitive transcription factor stimulated by oxidative stress, upon which it translocates to the nucleus and activates several antioxidant response elements (ARE)-regulated genes, including TrxR1 [38, 47, 48]. It is anticipated that auranofin causes the accumulation of overwhelming cellular levels of ROS that surpass the antioxidant buffering capacity of the cancer cells leading to their demise because of DNA damage [29, 47, 49–51]. Another important finding about auranofin that opens its use as an anticancer drug is the amplitude of cancers it may target considering that a large number of cancers carry a p53 gene mutation, while auranofin inhibits TrxR1 in a p53-independent manner [52].

Drugs that have been developed for other uses are also capable of inhibiting the TrxR1/Trx1 system. One is the case of cisplatin, a highly popular and first metal-based anti-cancer drug [53] that inhibits TrxR1 in an irreversible manner [54]. Cisplatin is a DNA-damaging agent known to cause cell death via direct toxicity towards the mitochondria, and by hybridizing the DNA forming DNA cross-links that are difficult to repair, leading to cell death [55, 56]. Another group of compounds that inhibit TrxR1 are histone deacetylase inhibitors (HDACi), which target cancer cells by their chromatin modifying effects [57]. One HDACi termed SAHA inactivates the function of Trx1 by binding to it, thereby leading to oxidative stress and induction of apoptosis [39]. Furthermore, in lung cancer cells, it was detailed that SAHA induced the down-regulation of Trx1, leading to the activation of ASK, which induces apoptotic cell death by triggering the ASK-JNK or ASK-p38 kinase pathways [58].

## Preclinical evidence of auranofin monotherapy eliciting anti-cancer effects

Increasing evidence on the preclinical mechanistic effects of auranofin against cancer cells was mounted in the last ten to fifteen years, particularly based on the capacity of the compound to increment the oxidative stress environment within the cancer cells. For instance, a study reveals that head and neck squamous carcinoma rely heavily on Trx1 for survival. This was confirmed by treating the head and neck cancer cells with auranofin plus or minus the ROS scavenger *N*-acetyl cysteine (NAC), which ameliorates ROS-induced DNA damage [59, 60]. Pre-treatment with NAC counteracted the cancer cell killing effects of auranofin, indicating that regulation of ROS is a primary mechanism used by head and neck cancers to multiply and spread [61]. In another study, auranofin inhibited the proliferation of mesothelioma cells in a dose-dependent manner; the anti-cancer effect was associated with caspase-independent apoptosis and necrosis, and increased ROS levels. The toxicity of ROS in these cells was demonstrated by the fact that NAC also prevented auranofin-induced lethality [62]. Of interest, the DNA damage caused by auranofin-induced ROS seems to be favored by increased membrane fluidity as shown in ovarian cancer cells with higher membrane fluidity (IGROV-1) being more sensitive to auranofin than ovarian cancer cells with lower membrane fluidity (OVCAR-5) [63]. Altogether, these evidences emphasize that ROS regulation plays a primary role in cancer cell survival and proliferation, and further supports the possible repurposing of auranofin against various cancers due to the pro-oxidative mechanism it triggers.

One of the major pathways that auranofin targets is the PI3K/AKT/mTOR pathway. This pathway is involved in the regulation of cell proliferation, apoptosis, and angiogenesis, and it is commonly associated with disease progression and tumorigenesis. Evidence shows that auranofin induces cytotoxicity in human pancreatic adenocarcinoma and non-small cell lung cancer via the inhibition of the PI3K/AKT/mTOR pathway [64]. The anti-cancer effect of auranofin was also analyzed in a human pancreatic adenocarcinoma in vivo model, a type of cancer in which pro-angiogenic factors, hypoxia-inducible factor 1 $\alpha$ (HIF-1 $\alpha$), and vascular endothelial growth factor (VEGF), are overexpressed [65]. This study revealed that auranofin inhibits pancreatic tumor growth at the primary tissue site, and inhibits metastasis in distant organs as well. Additionally, auranofin inhibits the cancer cell response to hypoxia, demonstrated by a decrease in HIF-1 $\alpha$ expression and VEGF secretion upon auranofin treatment under hypoxic conditions [65]. The authors conclude that this decreased expression of HIF-1 $\alpha$ induced by auranofin is possibly due to the inhibition of AKT, which normally helps stabilize HIF-1 $\alpha$ via VEGF production [65].

Another mechanism of action that auranofin uses to elicit cytotoxicity in cancer is by disrupting protein homeostasis. The action of auranofin in the inhibition of protein homeostasis was detected in HepG2 liver hepatocellular carcinoma cells and MCF-7 breast cancer cells [66]. In this study, auranofin induced apoptosis in HepG2 and MCF-7 cells by inhibiting the proteasome, a protein complex that degrades damaged or misfolded proteins [67]. In ovarian cancer cells, it was also shown via a proteomic analysis, that auranofin highly downregulated the expression of proteasome-related proteins [68]. The cytotoxic effect induced by auranofin has also been shown to be dependent on the inhibition of proteasome-associated deubiquitinases (DUBs), which are associated with cell growth and cancer progression [69]. These proteasome-associated DUBs are attractive therapeutic targets due to their role in regulating protein homeostasis within the cell. The inhibition of DUBs by auranofin results in the inhibition of tumor growth in mice with HepG2 and MCF-7 tumors [66]. Additionally, the cytotoxicity of auranofin by inhibition of proteasome-associated DUBs was also observed in chronic myeloid leukemia (CML) [70]. These findings are significant because auranofin can be considered one of the first DUB inhibitors already been in clinical use.

Aside from the evidence that auranofin targets different pathways within the cancer cell, the drug has been shown to target cancers of various genetic backgrounds. For instance, auranofin was shown to induce caspase-3-mediated apoptosis in human ovarian carcinoma SKOV-3 cells, which are deficient in the p53 tumor suppressor gene; this work also reported that auranofin toxicity was dependent on FOXO3 expression [71]. The efficacy of auranofin regardless of p53 functionality was also observed in p53 mutant refractory B-cell lymphoma, which happens to also carry a deletion in the tumor suppressor PTEN [72]. Another study using ovarian cancer cells demonstrated that the sensitivity of the cells to auranofin is enhanced by BRCA1 deficiency; BRCA1 is involved in DNA repair and in regulating the stability of anti-oxidant transcription factor Nrf2 via protein–protein interaction. BRCA1 deficient cells are usually more susceptible to oxidative stress. The BRCA1 deficient cells treated with auranofin had increased DNA double-strand breaks, while the overall lethality of auranofin was prevented by NAC, indicating ROS-mediated damage [73].

Another study highlights the efficacy of auranofin regardless of the sensitivity of the cancer cells to platinum drugs, one of the most widely used anti-cancer agents [56]. Auranofin induced cell death in a pair of sibling ovarian cancer cell lines that are either sensitive (OV2008) or resistant (OV2008/C13) to cisplatin [32]. The efficiency of auranofin against the cisplatin resistant cells was attributed to increased TrxR1 activity associated with the acquisition of resistance [32]. These findings signify that auranofin could be used to overcome platinum resistant diseases. For instance, we recently demonstrated that auranofin is equally potent in inhibiting the function, growth, and viability of high-grade serous ovarian cancer cells termed PEO1, which were obtained from a patient when platinum sensitive, than of PEO4 cells, which were obtained from the ascites of the same patient when she became platinum resistant following treatment [74–76]. We showed that auranofin-induced cytotoxicity was also associated with apoptotic cell death and required the induction of oxidative stress, as the lethality was prevented by the antioxidant NAC [76].

It is important to mention that novel TrxR1 inhibitors have been discovered eliciting similar mechanisms than auranofin against cancer cells. For instance, a small molecule MJ25, which was developed to increase the level of p53-dependent transactivation in malignant melanoma cells, was also identified as an irreversible TrxR1 inhibitor. In these cells, MJ25 and auranofin were both lethal at low concentrations and their effectiveness were ameliorated by supplementation of selenium, demonstrating the selenoprotein dependency of the lethal effect of the compounds [77]. In this study the authors also demonstrated that if they further depleted the cells of their antioxidant systems, but abrogating intracellular glutathione (GSH) levels with l-buthionine sulfoximide (BSO), co-treatment with MJ25 or auranofin led to complete cellular eradication [77] demonstrating that simultaneous depletion of the glutathione and TrxR antioxidant systems led to enhanced cytotoxicity [47, 48, 62, 78]. Very recently, it was shown in acute lymphoblastic leukemia (ALL), the most relevant pediatric cancer, that auranofin had a selective anti-cancer activity upon a screen of a library of 3707 approved drugs and pharmacologically active compounds; auranofin killed ALL cells via increasing ROS in the context of highly reduced levels of antioxidant glutathione [79].

Another compound that operates similarly to auranofin blocking the TrxR1/Trx1 system is piperlongumine, which is an alkaloid isolated from the fruit of the long pepper; results show that piperlongumine induces a lethal endoplasmic reticulum (ER) stress response by increasing the levels of ROS thus causing apoptosis [80]. Other new TrxR1 inhibitors result from the synthesis of hybrid compounds among azelaic acid (AZA) and organic arsenicals. One of these derivatives, termed A-Z2 [for *N*-(4-(1,3,2-dithiarsinan-2-yl) phenyl)-azelamide], shows stronger activity against TrxR1 activity in acute myeloid leukemia (AML) cell lines than did AZA or arsenicals separately. The compound activates the intrinsic apoptotic pathway by selectively targeting TrxR1/Trx1 and indirectly inhibiting NF-kB; the compound also demonstrated efficacy in vivo against a patient-derived xenograft (PDX) AML model [81].

## Anti-cancer effects of auranofin derivatives

Various gold (I) phosphine complex derivatives were developed since the anticancer properties of auranofin were revealed. These compounds incorporate a variety of ligands aiming to increase the anticancer activity of the complex, but without removing the triethylphosphine moiety, which is critically important to maintain the cytotoxic potency of the molecule [2, 82]. Thus, a gold (I) complex with chelated diphosphines such as [Au(dppe)_2_]Cl was synthesized and showed high toxicity against various cancer types [83]; yet, this compound never reached the clinic due to unfavourable preclinical toxicity studies [2] Next, a related compound that included the propyl-bridged 2-pyridyl phosphine ligand (d2pypp) identified as [Au(d2pypp)_2_]^+^ was developed with the idea of enhancing the reactivity against TrxR while increasing its accumulation in the mitochondria [84]. This compound showed selectivity against breast cancer cells while sparing normal breast cells [85]. Other derivatives with anticancer effect are the lipophilic cationic gold (I) phosphine complex [Au(dppp)(PPh3)Cl] [86], the hydrophilic four-coordinate complex [Au(P(CH_2_OH_3_)_4_]Cl] [87], and the highly lipophilic cationic Au (I) *N*-heterocyclic carbene complexes (NHC) of the form [R_2_Im)_2_Au]^+^ [88]. Two structurally related gold (I)-*N*-heterocyclic carbenes complexes tested in ovarian cancer cells show, similarly to auranofin, a potent inhibition of TrxR, yet display differences in the magnitude of protein regulation as assessed by comparative proteomic analysis; the dicarbene derivative showed many more proteins regulated than its monocarbene counterpart, while displaying higher antiproliferative properties in three different ovarian cancer cell lines [89].

Potent and selective TrxR inhibition was also achieved by a series of linear gold (I) compounds resembling auranofin, all containing the [Au(PEt_3_)]^+^ synthon with different ligands—Cl^−^, Br^−^, cyanate, thiocyanate, ehtylxanthate, diethyldithiocarbamate or thiourea—and having antiproliferative effects towards multidrug-resistant and platinum-resistant cancer cells [90]. More recently, the derivative of [Au(PEt3)]^+^ with iodide or chloride ligands (also termed Et3PAuI and Et3PAuCl) were shown to have high cytotoxicity against colorectal cancer cells in vitro and in vivo and with a potency similar to that of auranofin, while suggesting that the presence of the thiosugar moiety in auranofin is not mandatory for the pharmacological action [91]. Moreover, in an orthotopic mouse model of ovarian cancer, it was demonstrated that not only replacement of the thiosugar in auranofin is not required for its pharmacological function, but that its iodide analogue, the idodide(thiethylphosphine)gold I complex (or Et3PAuI), has much higher anticancer potency than that of auranofin while being well tolerated [92]. Such replacement of the thiosugar with iodine involves a higher reactivity of the molecule towards certain amino acids such as histidine, cysteine, methionine, and selenocysteine, which could explain the enhanced antitumoral activity of this iodide analogue of auranofin [93]. Furthermore, Et3PAuI demonstrated its efficacy against ovarian cancer cells resistant to platinum, yet it retains the cross-resistance towards auranofin in auranofin-resistant ovarian cancer cells [94]. Other analogues of auranofin were synthesized with the general formula Au(Pet_3_)X, where X is a pseudohalide group (cyanide, thiocyanate, or azide) that replaces the thiosugar in auranofin; the compounds have biological activity yet showed lesser cytotoxicity than auranofin towards colon cancer cells [95].

## Auranofin anti-cancer activity in combination treatments

In human lung cancer, auranofin induces cytotoxicity via ROS production in stem cell-like cancer cells, a side-population (SP) that has high expression of ATP-binding cassette transporter 2 (ABCG2) [96]. SP cells are significant because they are able to develop drug resistance due to increased drug export via ABCG2. Interestingly, it was found that auranofin inhibits ABCG2 function by depleting cellular ATP via inhibition of glycolysis [96]. The disruption in cellular ATP levels by auranofin leads to the inhibition of ABCG2 function in drug export, preventing the SP cells from developing drug resistance. This suggests that auranofin can be used to improve the efficacy of other drugs by inhibiting the drug resistant mechanism of SP cell populations. This is demonstrated in human lung cancer cells, in which auranofin synergizes with adriamycin to kill the cancer cells more potently than when the cells are treated with adriamycin alone [96]. Also in lung cancers auranofin was shown to be highly efficient when the glutathione antioxidant system, which usually complements the thioredoxin system, is compromised; this is a case of synthetic lethality in which auranofin is highly cytotoxic by inhibiting TrxR in a background of glutathione deficiency [97].

Of interest, cancer cells can develop resistance to auranofin by reduced drug accumulation caused by the dysregulation of influx and efflux drug transporters, as demonstrated in ovarian cancer cells that were made approximately 20-fold resistant to the gold I complex upon stepwise exposure of a parental cell line to increasing auranofin concentrations during an 8-month selection period [98]. The development of resistance to auranofin seems to be very specific as the resistant cells retained sensitivity to other investigational gold compounds, as well as to approved chemotherapeutics such as oxaliplatin, vinblastine, doxorubicin, etoposide, and paclitaxel [98].

In malignant B-cells, one study demonstrates that when exposed to high concentrations of l-ascorbate, the cells die due to autoxidation and generation of H_2_O_2_; this mechanism is ameliorated by the counterbalancing activation of the Trx1 system. However, when cells were exposed to l-ascorbate in combination with auranofin, the H_2_O_2_ scavenging capacity of malignant B-cells was depleted. Of interest, in this combination therapy, the lethality was associated with the accumulation of radical hydroxyl (ˑOH) [99], generated by the catalysis of Fe^2+^ into Fe^3+^ via the Fenton reaction triggering iron-dependent cytotoxicity or ferroptosis [100]. This mechanism of toxicity induced by auranofin was also observed in human retinal pigment epithelial cells, in which auranofin-induced lethality was prevented by the presence of the ferroptosis inhibitor ferrostatin-1 [101].

In lung cancer cells, auranofin was shown to interact with the natural inhibitor of TrxR1, selenocysteine, both in vitro and in vivo, by enhancing the accumulation of ROS [102]. Likewise, auranofin synergizes with another Trx1 inhibitor, piperlongumine, in killing gastric cancer cells in association with ROS-mediated ER stress response and mitochondrial dysfunction. The synergistic lethality of auranofin and piperlongumine was also observed in vivo in a xenograft tumor model, and is consequence of the overwhelming ROS producing capacity of the cells when the drugs are combined [103]. Another case in which the toxicity of auranofin is associated with the overproduction of ROS is in both platinum sensitive OV2008 ovarian cancer cells and their resistant counterparts, OV2008/C13, when the gold complex is combined with either selenite or tellurite [104]. Such strategy of raising intracellular ROS to levels that cannot be managed by the antioxidant systems was demonstrated in MCF-7 breast cancer cells. In this case, a synergistic interaction was found between auranofin and mesupron blocking the growth of MCF-7 cancer cells. Mesupron is a small molecule that inhibits urokinase-type plasminogen activator (uPA) and causes mitochondrial dysfunction. When associated to the overproduction of ROS triggered by auranofin, the cells undergo synergistic apoptosis mediated by caspase-3 activation, and downregulation of antiapoptotic mitochondrial factors including BCL2, BCL-xL, and MCL1. All these effects are associated with disruption of the mitochondrial potential [105] and mediated by the generation of ROS, as the toxicity was abrogated by NAC [106]. Along the same line of research, it was reported in colon cancer in vitro and in vivo that auranofin synergises with the anti-inflammatory drug celecoxib via a mechanism that includes severe ROS production and oxidative stress and disturbance of mitochondrial redox homeostasis leading to a depletion of ATP [107]. Finally, a report shows that oxidative stress can be over-powered in A549 lung cancer cells by the combination of auranofin and KU55933, an inhibitor of the serine/threonine kinase ATM that is involved in sensing DNA damage upon DNA double strand breaks [108]. The toxicity of the combination therapy was rescued by ROS scavengers, demonstrating once again the critical role of redox homeostasis to maintain the wellbeing of cancer cells that operate with dangerously high background levels of ROS [49].

An interesting mechanism of toxicity was reported in brain tumor cells. In this case auranofin was used to kill glio- and neuroblastoma cells by inhibiting TrxR1 in combination with CyPPA [109], which is an opener of the small-conductance calcium-activated potassium channels [110]. The toxicity of the combination of auranofin and CyPPA results in massive mitochondrial damage, which was not only observed in two-dimensional cell cultures, but also was recapitulated in glioblastoma neurospheres. Another case in which mitochondrial stress was reported upon the action of auranofin is in breast cancer cells when auranofin was combined with the mitogen-activated protein kinase (MAPK) inhibitor trabetinib. In this case, MCF-7 cells treated with the combination auranofin/trabetinib underwent apoptosis associated with activation of executer caspases-3/7, activation of the p38 MAPK signaling pathway, and translocation of apoptosis inducing factor (AIF) from the mitochondria to the nucleus [111].

Using a high-throughput viability screen and reliable in silico data allowed the rational identification of two pathways that can lead to synergistic interaction towards cell death in ovarian cancer. The study discovered that the TrxR inhibitor and ROS inducer auranofin synergized in the killing of ovarian cancer cells with the heat-shock protein 90 (HSP90) chaperone antagonist, AUY922 [112].

Finally, there is even a case in which auranofin synergizes with a combination treatment leading to a triplet of molecules targeting the same cancer cells. Auranofin was reported to interact in a synergistic manner with a combination of erlotinib [an inhibitor of activated epidermal growth factor receptor (EGFR)] killing non-small cell lung carcinoma cells which have been restored of tumor suppressor candidate 2 (TUSC2, also known as FUS1). In this case, the combination of TUSC2 restoration with erlotinib generates the vulnerability to allow auranofin-mediated toxicity [113]. A summary of preclinical studies done with auranofin and other compounds is presented in Table 1.

**Table 1:** Cytotoxicity of auranofin in combination treatments against different cancers

Drug combination	Mechanism(s) of action	Cancer	Cells/animal models	References
AUF + Celecoxib	ROS mediated inhibition of hexokinase and glycolysis Disruption in mitochondrial oxidative phosphorylation	Colon	HCT116, HT-29, DLD-1, DLD-1 tumors in nude mice	[107]
AUF + Adriamycin	ROS-mediated inhibition of glycolysis, ATP production, and ABCG2 transporter expression Decreased drug resistance	Lung	A549, NCI-H460, A549 tumors in athymic nude mice	[96]
AUF + l-ascorbate	Iron-dependent inhibition of H_2_O_2_ scavenging capacity H_2_O_2_-dependent cell death DNA damage	Burkitt Lymphoma Chronic lymphocytic leukemia	Malignant B cells Raji and Mec-1, human CLL cells	[99]
AUF + Piperlongumine	ROS-mediated ER stress and mitochondrial dysfunction Caspase-3/PARP1-dependent apoptosis	Gastric	BGS-823, SGC-7901, KATO III	[103]
AUF + Mesupron	ROS-mediated caspase-3-dependent apoptosis AIF nuclear translocation	Breast	MCF-7	[106]
AUF + KU55933	ROS-mediated oxidation of antioxidant protein PRDX1/3 via TrxR and ATM inhibition	Lung	A549, MLF	[108]
AUF + CyPPA	Cell death and mitochondrial damage via SK channel activation	Glioblastoma Neuroblastoma	SK-N-AS, U251	[109]
AUF + Trametinib	Caspase-3/7-dependent apoptosis AIF nuclear translocation via p38/MAPK phosphorylation	Breast	MCF-7	[111]
AUF + Selenocysteine	ROS-mediated apoptosis via inhibition of PI3K/AKT and MEK/ERK pathways DNA damage	Lung	A549 and A549 tumor xenografts in mice	[102]
AUF + Selenite	ROS-mediated inhibition of TrxR, GPx, and GR Apoptosis	Ovarian	2008 (cisplatin-sensitive) and C13* (cisplatin-resistant)	[104]
AUF + AUY922	Cell death via ROS and HSP90 inhibition	Ovarian	A1847, OVCAR4, PEO4, SKOV3, OVCAR8	[112]
AUF + Erlotinib + TUSC2	NRF2-mediated oxidative stress ROS-mediated apoptosis and inhibition of colony formation DNA damage Increased animal survival	Lung	Wild-type EGFR NSCLC Calu-3, Calu-6, and H522, H157, H1299, human NSCLC H1299 tumors in mice	[113]

*AUF* auranofin, *ROS* reactive oxygen species, *ATP* adenosine triphosphate, *ABCG2* ATP binding cassette subfamily G member 2, *ER* endoplasmic reticulum, *PARP* poly(ADP-ribose) polymerase-1, *AIF* apoptosis-inducing factor, *PRDX1/3* peroxiredoxin 1, *TrxR* thioredoxin reductase, *ATM* ataxia-telangiectasia mutated kinase, *SK* small-conductance calcium-activated potassium channel (SK/K_Ca_), *PI3K* phosphatidylinositol 3-kinase, *GPx* glutathione peroxidase, *GR* glutathione reductase, *TUSC2* tumor suppressor gene TUSC2, *EGFR* epidermal growth factor receptor, *NSCLC* non-small cell lung cancer, *NRF2* nuclear factor erythroid 2-like factor 2, p53 tumor suppressor p53, *HSP90* heat shock protein 90

## Summary of mechanisms of action of auranofin as an anti-cancer agent

**Fig. 2:**
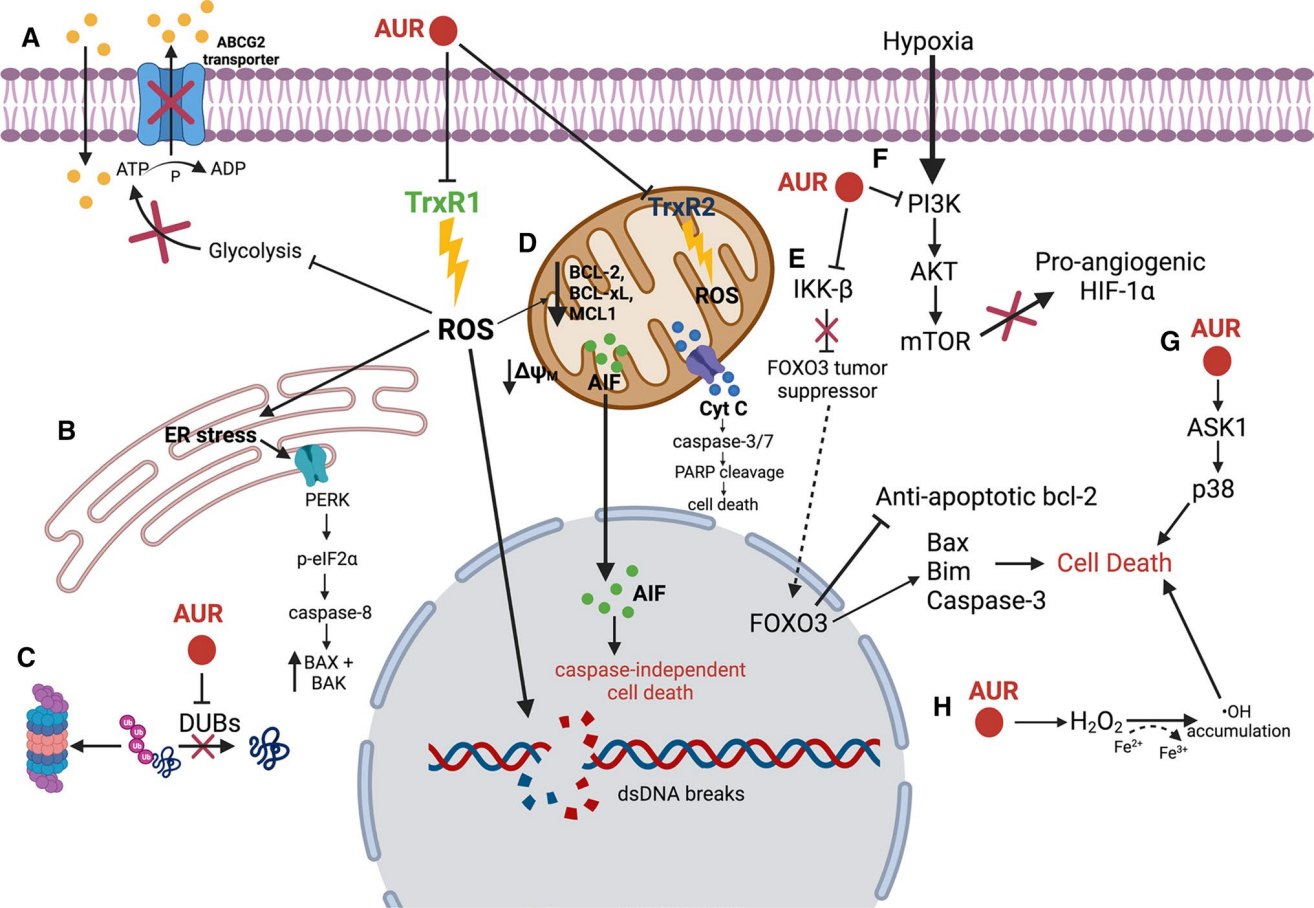
Auranofin (AUR) inhibits the anti-oxidant enzymes thioredoxin reductase 1 (TrxR1) and 2 (TrxR2) resulting in an increase in the level of intracellular reactive oxygen species (ROS), mitochondrial permeability, and DNA damage. **A** AUR inhibits glycolysis resulting in reduced ATP levels and inhibition of the function of drug transporter, ABCG2, preventing development of drug resistance. **B** AUR-mediated ROS production causes ER stress and PERK activation, leading to cell death. **C** AUR inhibits the function of deubiquitinases enzymes (DUBs) in protein homeostasis and induction of tumor growth. **D** Increased ROS induced by AUR causes decreased membrane potential in the mitochondrial membrane, resulting in a decrease in anti-apoptotic proteins, caspase-dependent cell death, and translocation of apoptosis-inducing factor (AIF) into the nucleus to trigger caspase-independent cell death. **E** AUR inhibits the IKK-β signaling pathway, which normally induces FOXO3 tumor suppressor degradation. The inhibition of IKK-β by AUR allows the nuclear translocation of FOXO3, activating proapoptotic proteins, resulting in cell death. **F** AUR inhibits the PI3K/AKT/mTOR pathway, resulting in the inhibition of pro-angiogenic factors like HIF-1α, preventing tumorigenesis. **G** AUR activates ASK1 leading to p38-mediated cell death. **H** AUR promotes the conversion of H_2_O_2_ to ˑOH via the Fenton reaction, resulting in the induction of cell death

All mechanisms of action previously described for auranofin as an anti-cancer agent are summarized below in Fig. 2.

## Clinical translational advances on auranofin in cancer

Due to the promising evidence in the literature on the use of auranofin as an anti-cancer agent, the drug has been enrolled in phase I/II clinical trials to treat patients with the following diseases: chronic lymphocytic leukemia, non-small cell lung cancer or small cell lung cancer, and ovarian, peritoneal, and fallopian tube cancers (see United States National Library of Medicine; www.clinicaltrials.gov, trial numbers NCT01419691, NCT01737502, NCT01747798, and NCT03456700). The aim of these clinical trials is to measure the overall response rate to auranofin, to address any adverse effects of the drug, and to assess the survival rate of the patients following exposure to the treatment. Additionally, these clinical trials include patients with metastatic or recurrent disease, in which auranofin is being used as a possible consolidation therapeutic agent to improve overall survival rate. However exciting these clinical trials are, their outcomes have yet to be reported.

## New viewpoint of research with auranofin against cancer: the link with platinum-based chemotherapy, immunogenic cell death, and immunotherapy

**Fig. 3:**
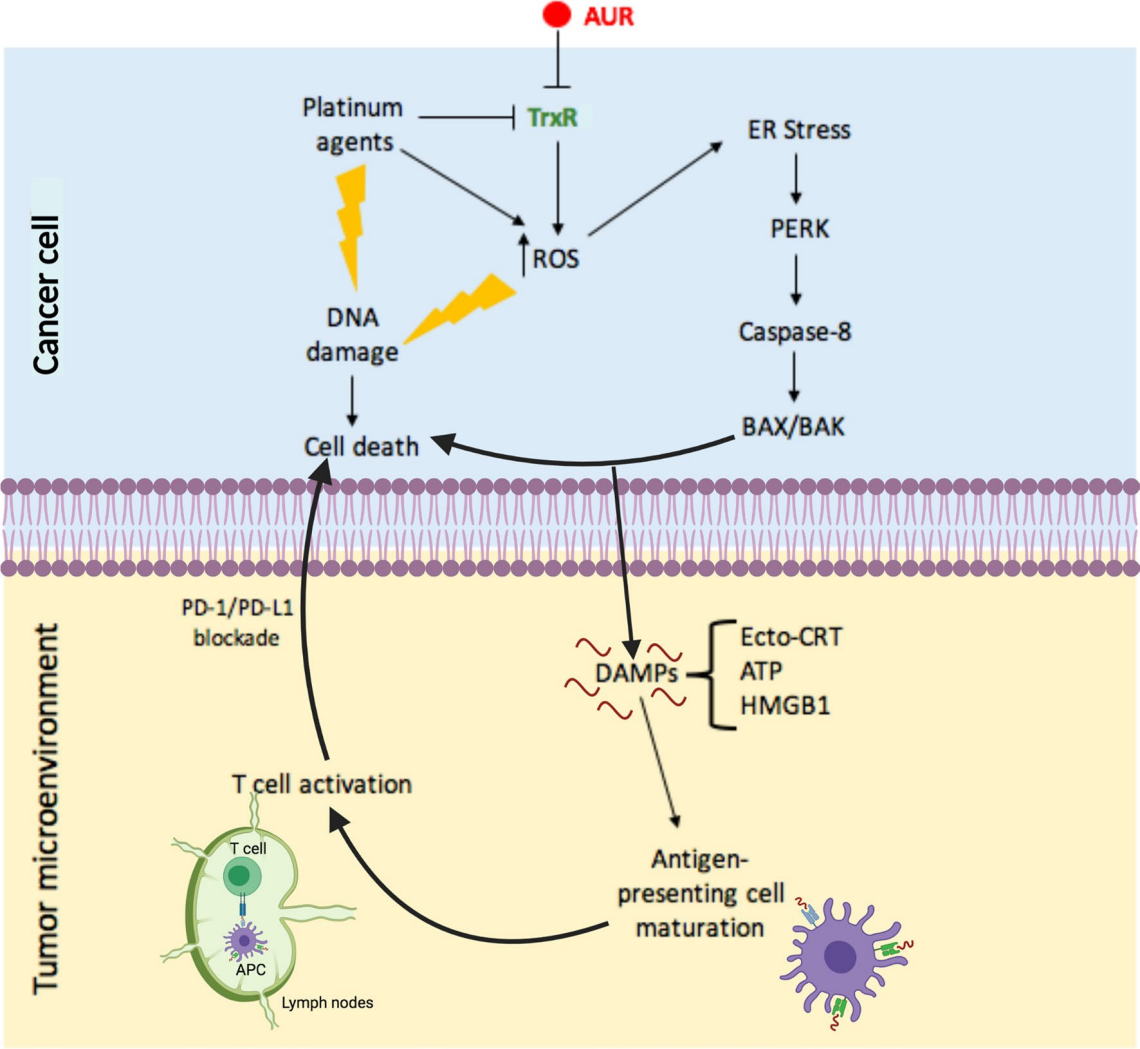
Targeting cancer cells with a combination of auranofin (AUR) and a platinum agent in the context of the tumor microenvironment. AUR and platinum agents inhibit the function of TrxR, inducing an overproduction of reactive oxygen species (ROS), mitochondrial permeability, and DNA damage, resulting in cancer cell death. Accumulation of intracellular ROS induces ER stress, activation of PERK and caspase-8, and the upregulation of pro-apoptotic proteins BAX and BAK, further potentiating cell death. This pathway triggers the release of danger or damage associated molecular patterns (DAMPs) such as calreticulin (ecto-CRT), high mobility group box 1 (HMGB1) protein and ATP into the tumor microenvironment where they collectively activate antigen presenting cells (APCs). Mature APCs migrate into the lymph nodes and present tumor antigens to immature T cells, which develop into CD8+T cells with anti-cancer cytotoxic activity. The inhibition of the interaction between PD-1 on T cells and PD-L1 on cancer cells prevents the neutralization of T cells by cancer cells, maintaining T cell cytotoxicity and favoring tumor cell death. This release of DAMPs into the tumor microenvironment via ER stress and the consequent activation of T cells is known as immunogenic cell death or ICD

We propose that a potential synergism of platinum agents with auranofin would have two folds: (1) irreversible toxicity caused by accumulation of ROS and irreparable DNA damage; and (2) simultaneous induction of immunogenic cell death (ICD) with negative consequences for the survival of the tumor (Fig. 3).

First, the toxicity of the widely used platinating agent cisplatin is proportional to the level of expression of TrxR1 [114], and cisplatin also inhibits TrxR1 with high specificity [54]. Furthermore, cancer cells that operate with increased oxidative stress are more vulnerable to damage by further ROS insults induced by exogenous agents [115] when compared to normal cells [49]. Based on this background, it is tempting to hypothesize that the oxidative stress caused by the combination auranofin/cisplatin will be high enough to disrupt the antioxidant capacity of cancer cells and to cause sufficient DNA damage that the cells will not be able to repair, resulting in cell death.

Second, gold compounds were suggested to be able to induce ICD [116] based on the observations that different gold derivatives caused ROS-mediated necroptosis in colon cancer cells [117], and triggered ER-stress mediated apoptosis and autophagy in non-small cell lung carcinoma [118]. However, it was not until very recently that auranofin was reported, in non-small cell lung cancer cells, to actually trigger the release of biomarkers that are characteristics of ICD [119]. This is a type of cell death involving an atypical apoptotic process, in which the cells, while dying, release a series of molecules to the tumor microenvironment. These ‘alarm’ molecules, also known as danger signals, alarmins, or more precisely damage-associated molecular patterns (DAMPs), makes such type of apoptotic cell death ‘immunogenic’ because they promote an inflammatory response. DAMPs attract innate immune cells, especially antigen presenting cells (APCs) such as dendritic cells, leading to the phagocytosis of the dying cells followed by transportation to the lymphatics where tumor antigens are presented to T cells [9, 116, 120–123]. The most critical DAMPs released during ICD are calreticulin (CLR), ATP, and high-mobility group box 1 (HMGB1) protein [124]. The mechanism whereby CLR moves from its original site—the ER—to the plasma membrane, requires the ER stress associated phosphorylation of the eukaryotic translation initiation factor eIF2α, resulting in arrest of mRNA translation, activation of caspase-8, upregulation of pro-apoptotic BCL-2 family members BAX and BAK, the transport of CRT to the Golgi apparatus, and exocytosis of CRT-containing vesicles [125]. The presentation of CRT on the surface of the plasma membrane (a.k.a. ecto-CRT) occurs very early during the process of ICD, even before lipid molecules carrying phosphatidylserine residues are exposed to the outer leaflet of the plasma membrane of cells undergoing apoptosis [126]. The release of ATP and the non-chromatin DNA chaperone HMGB1 protein occurs later on in the cell death process, and is mediated via an autophagy-associated mechanism [9, 127]. All three molecular components are essential for the promotion of maturation of APCs [121].

We anticipate that because auranofin causes ICD in non-small cell lung carcinoma [119], the overall lethality would be increased by the presence of one of the DNA damaging platinating agents that constitute the standard of care for this cancer [128, 129]. It is tempting to speculate that other platinum-responsive cancers, such as cervical, head and neck, testicular, bladder, and ovarian to mention some ([55]), may carry the basic molecular backgrounds to also be responsive to auranofin-mediated ICD. It is imperative that the scientific community investigates in detail whether the lethal effect of auranofin towards platinum-sensitive and resistant cancers involve all or some of the components of the ICD pathway. This knowledge may create opportunities for combining auranofin with platinating derivatives known not to be per se effective inducers of ICD, such as cisplatin or carboplatin [130, 131]. Of the three platinum derivatives, cisplatin, carboplatin, and oxaliplatin, that are clinically approved worldwide against cancer, the latter is the only one demonstrated to be highly efficient in inducing ICD, yet it is only widely used to treat mostly colorectal carcinomas or platinum-sensitive ovarian cancer patients that had become allergic to carboplatin [132–135]. Thus, the capacity of auranofin to complement cisplatin and carboplatin in effectively inducing ICD may be efficient against many cancers. Auranofin and cisplatin, or carboplatin, may interact and act as a ‘vaccine’ in which cancer cells dying by ICD, via DAMPs, activate APCs; these, in turn, present the cancer antigens to T cells in the lymph nodes, leading to the generation of active tumor specific CD8+T cells with the capacity to attacking and killing other cancer cells within the tumor microenvironment [9, 120, 123, 124].

In another exciting study, in this case in breast cancer, it was shown that auranofin was highly effective in causing cell death and impairing the growth of triple negative breast cancer cells grown as spheroids. In this work, also auranofin was effective in vivo in patient-derived tumor xenografts by inhibiting TrxR1 activity and increasing CD8+T cell infiltration [136]. All these effects occurred in combination with the upregulation of PD-L1, a member of the PD-1/PD-L1 immune checkpoint [136]. As expected, combination of auranofin with an anti-PD-L1 antibody synergistically impaired the growth of syngeneic 4T1.2 primary tumors [136]. PD-L1 is expressed on the surface of cancer cells and usually engages with PD-1 expressed on the surface of T cells to neutralize their activity [137]. Additionally, PD-L1 is overexpressed in many cancers and is associated with tumor chemoresistance and poor clinical outcomes [138]. Furthermore, anti-PD-L1 therapy was effective in synergizing with the ICD properties of oxaliplatin in hepatocellular carcinoma in vivo [139]. Thus, adding anti-PD-1 or anti-PD-L1 antibodies on the top of auranofin/cisplatin combination therapy seems a rational combinational therapeutic approach. This idea is further supported by recent literature showing that breast cancer tissues were sensitive to mifepristone—an antiprogestin and antiglucocorticoid agent [140], which was able to induce ICD and thus synergize with PD-L1 blockade [141]. We previously reported that the anti-cancer effects of mifepristone [142, 143] were associated with the induction of proteotoxic ER stress [144], a pathway needed to be active for ICD to succeed.

Finally, another important avenue to pursue if auranofin and platinum agents synergize in killing cancer cells is to develop a therapeutic approach by synthesizing a hybrid compound between cisplatin and auranofin. Such hybrid compound would have higher lethality than the individual drugs against cancer cells resistant to standard platinum-based therapy at the time of recurrence. Hybrid compounds simultaneously targeting different points of signaling networks and various structures within cancer cells were explored extensively in recent years [145]. It has become clear that it is very difficult to achieve desirable chemotherapeutic effects in the treatment of advanced cancers using single drug therapy. Thus, a multi-target approach utilizing a hybrid molecule should provide greater therapeutic anticancer benefits and better safety while reducing the risk of developing drug resistance [145, 146]; we anticipate that a hybrid drug between cisplatin and auranofin will achieve such a goal.

## Concluding remarks

Auranofin is considered safe for human use in treating rheumatoid arthritis; thus, this gold derivative can reach the clinic for other diseases relatively quickly and at a low cost, taking into account that the drug has a well-known toxicity profile [147]. Auranofin and other gold-related compounds emerge as highly promising agents to be repurposed for cancer therapy mainly in combination with platinum derivatives thus encompassing a large number of tumor types. Synergistic interaction between auranofin and platinating agents may not only involve unrepairable ROS-induced DNA damage, but also induction of an immune response due to the capacity of auranofin to induce ICD. This places the combination auranofin-platinum combination therapy, working either as complementary drugs, or via a hybrid molecule, within the field of immune cancer therapies. As it is clear that combination treatments are most likely the reason whereby cancer will be hopefully under control in the near future, we suggest including as part of a multiplex therapy, in addition to auranofin and a platinum agent, immune checkpoint inhibitors to increase the possibility of transforming a lethal disease into a controllable chronic one.

## Abbreviations

AIF: Apoptosis inducing factor
ALL: Acute lymphoblastic leukemia
AML: Acute myeloid leukemia
AKT: Protein kinase B
APC: Antigen presenting cells
ARE: Antioxidant response element
ASK: Apoptosis signaling kinase
ATM: Ataxia telangiectasia mutated
AUF: Auranofin
BCL-2: B-cell lymphoma 2
BCL-xL: B-cell lymphoma-extra large
BRCA1: Breast cancer gene 1
BSO: l-buthionine sulfoximide
CML: Chronic myeloid leukemia
CRT: Calreticulin
DAMPs: Danger- or damage-associated molecular patterns
DMARS: Disease-modifying antirheumatic drugs
DUBs: Deubiquitinases
EGFR: Epidermal growth factor receptor
eIF2α: α-Subunit of eukaryotic initiation factor 2 (eIF2)
ER: Endoplasmic reticulum
FOXO3: Forkhead box O3
GSH: Glutathione
HDACi: Histone deacetylase inhibitor
HIF1α: Hypoxia-inducible factor 1 alpha
HMGB1: High mobility group box 1
H_2_O_2_: Hydrogen peroxide
HSP90: Heat-shock protein of 90 kDa
ICD: Immunogenic cell death
IKK: IκB kinase
IL-6: Interleukin-6
IL-8: Interleukin-8
JAK: Janus kinase
JNK: C-JUN N-terminal kinase
MAPK: Mitogen-activated protein kinase
MCL-1: Myeloid cell leukemia 1
mTOR: Mammalian target of rapamycin
NAC: *N*-Acetyl cysteine
NADPH: Nicotinamide adenine dinucleotide phosphate
NF-kB: Nuclear factor kappa B
NSAIDs: Nonsteroidal anti-inflammatory drugs
ˑOH: Hydroxyl radical
PI3K: Phosphoinositide 3-kinase
PD1: Programmed cell death protein 1
PD-L1: Programmed death-ligand 1
PDX: Patient-derived xenograft
PERK: Protein kinase R (PKR)-like endoplasmic reticulum kinase
PTEN: Phosphatase and tensin homolog
ROS: Reactive oxygen species
SP: Side population
TNF: Tumor necrosis factor
TP53: Tumor suppressor protein p53
Trx: Thioredoxin
TrxR: Thioredoxin reductase
TUSC2: Tumor suppressor candidate 2
uPA: Urokinase-type plasminogen activator
UPS: Ubiquitin proteasome system
VEGF: Vascular endothelial growth factor
